# Secretory glands in cercaria of the neuropathogenic schistosome *Trichobilharzia regenti *- ultrastructural characterization, 3-D modelling, volume and pH estimations

**DOI:** 10.1186/1756-3305-4-162

**Published:** 2011-08-19

**Authors:** Anna Ligasová, Jana Bulantová, Ondřej Šebesta, Martin Kašný, Karel Koberna, Libor Mikeš

**Affiliations:** 1 Institute of Experimental Medicine, Academy of Sciences of the Czech Republic 14220 Prague 4, Czech Republic; 2 Department of Parasitology, Faculty of Science, Charles University in Prague, Viničná 7, 12844 Prague 2, Czech Republic; 3 Laboratory of Confocal Microscopy, Faculty of Science, Charles University in Prague, Viničná 7, 12844 Prague 2, Czech Republic

## Abstract

**Background:**

Cercariae of schistosomes employ bioactive molecules for penetration into their hosts. These are released from specialized unicellular glands upon stimuli from host skin. The glands were previously well-described in the human pathogen *Schistosoma mansoni*. As bird schistosomes can also penetrate human skin and cause cercarial dermatitis, our aim was to characterize the architecture and ultrastructure of glands in the neurotropic bird schistosome *Trichobilharzia regenti *and compare it with *S. mansoni*. In the context of different histolytic enzymes used by these two species, we focused also on the estimations of gland volumes and pH in *T. regenti*.

**Results:**

The architecture and 3-D models of two types of acetabular penetration glands, their ducts and of the head gland are shown here. We characterized secretory vesicles in all three gland types by means of TEM and confirmed accuracy of the models obtained by confocal microscopy. The results of two independent approaches showed that the glands occupy ca. one third of cercarial body volume (postacetabular glands ca. 15%, circumacetabular 12% and head gland 6%). The inner environment within the two types of acetabular glands differed significantly as evidenced by dissimilar ability to bind fluorescent markers and by pH value which was higher in circumacetabular (7.44) than in postacetabular (7.08) glands.

**Conclusions:**

As far as we know, this is the first presentation of a 3-D model of cercarial glands and the first exact estimation of the volumes of the three gland types in schistosomes. Our comparisons between *T. regenti *and *S. mansoni *implied that the architecture and ultrastructure of the glands is most likely conserved within the family. Only minor variations were found between the two species. It seems that the differences in molecular composition have no effect on general appearance of the secretory cells in TEM. Fluorescent markers employed in this study, distinguishing between secretory vesicles and gland types, can be useful in further studies of mechanisms used by cercariae for host invasion. Results of the first attempts to estimate pH within schistosome glands may help further understanding of regulation of enzymatic activities present within the glands.

## Background

Among several species of trematode cercariae penetrating the skin of vertebrate hosts, schistosomes are of particular interest as the causative agents of human disease (schistosomiasis - syn. Bilharziasis). The morphology and structure of penetration glands has been described in detail in *Schistosoma mansoni *cercariae. These glands are composed of five pairs of large secretory cells located in the vicinity of the ventral sucker (acetabulum); according to their position towards the sucker, ultrastructure and composition they have been divided into two groups. Three pairs have been originally designated as postacetabular and two as preacetabular [1], although the position of the latter is rather circumacetabular as generally accepted and shown in this paper (therefore this term will be used). This arrangement seems to be conserved among all schistosomes checked so far, e.g. [2-6]. The fine ultrastructure and development of *S. mansoni *acetabular glands has been described previously by several authors [7-10] using transmission electron microscopy (TEM).

The chemical composition of the acetabular glands allows staining by different histological dyes. In *S. mansoni*, circumacetabular glands are acidophilic and eosinophilic and can be stained by dyes with affinity for calcium such as alizarin and purpurin - e.g. [1,11]. Further experiments confirmed a high concentration of this bivalent cation in specific vesicles within circumacetabular glands of *S. mansoni *cercariae [12-14]. The ocurrence of calcium was also confirmed in circumacetabular glands of two schistosomes parasitizing birds - *Trichobilharzia regenti *and *T. szidati *[11]. The postacetabular glands are basophilic and can be stained after fixation with lithium carmine, aniline blue, thionin, toluidine blue etc. [1]. They gave positive results with periodic acid-Schiff (PAS) reaction, thus indicating the presence of reducing saccharides, probably in the form of polysaccharides, glycoproteins or glycolipids [2,15-17]. Oxidized apomorphine has been used for differentiation of acetabular gland types in various schistosomes - in *S. mansoni *and *T. szidati *postacetabular glands are stained dark green, whereas circumacetabular tend to be yellow-orange and red-brown, respectively. Slightly different patterns were observed among the studied schistosome species, probably reflecting differences in gland environment [11,18].

Another gland type of schistosome cercariae occuring within the muscular head organ is the head gland. In *S. mansoni*, this appears to be a relatively large unicellular entity consisting of a fundus tapering into a system of multiple ducts that open into the tegument at the anterior end of cercarial body [19,20]. However, some authors speculate that it may consist of two cells [10]. Although the exact function of the head gland is not known, it has been hypothesized that it provides material (phospholipids) for repair and reorganization of cercarial tegument injured during penetration [20] or that its secretions may be involved in the process of penetration or immune modulation of the host [21].

Bioactive molecules from schistosome acetabular penetration glands have been extensively studied since the 1950's, mainly in terms of their histolytic (peptidase) activities (for review see e.g. [22]). Only recent methods of molecular biology/genomics and proteomics/mass spectrometry have enabled better insight into the protein composition of acetabular gland secretions - e.g. [23-26].

The analyses focused on proteolytic enzymes of various schistosomes revealed differences in the types of enzymes used for host invasion by cercariae of particular species - e.g. *Schistosoma japonicum *and *Trichobilharzia *spp. seem to use cysteine peptidases in contrast to *S. mansoni, S. haematobium *and *Schistosomatium douthitti *employing serine peptidases for penetration [11,27-30]. This brought further questions about the range of possible variations in general composition of schistosome gland secretions (enzymes, other proteins and bioactive molecules) and the physico-chemical environments (e.g. pH, ionic strength) within compartments presented by particular penetration gland cell types.

Our study was focused on the architecture, ultrastructure and some other features of penetration glands employed by cercariae of the neuropathogenic bird schistosome *Trichobilharzia regenti *for invasion of its hosts. This schistosome has a unique route of migration within both specific (anatid birds) and nonspecific incidental hosts (mammals); shortly after entering the skin, schistosomula migrate through peripheral nerves to the CNS causing neuromotor disorders and paralyses [31]. Furthermore, it is able to cause cercarial dermatitis in humans (swimmers' itch). Therefore, the aim of our study was to provide a detailed ultrastructural and morphological picture of the glands of *T. regenti *cercaria and make some comparisons with the previously published data on the human pathogen *Schistosoma mansoni*. In addition, volumes of and pH within particular types of penetration glands were measured employing fluorescent probes and confocal microscopy.

## Results

### Gland architecture and 3-D modelling

**Figure 1:**
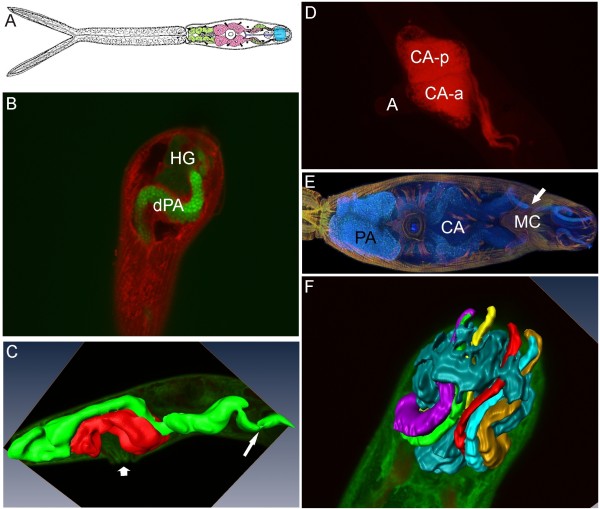
**Glands of the cercaria of *T. regenti***. **A**, schematic figure of the cercaria with highlighted glands; postacetabular glands in green, circumacetabular glands in pink, head gland in blue. **B**, z-section from CM of cercarial head organ stained with Alexa Fluor^® ^488 and Cy3-azide; secretory vesicles of postacetabular ducts express bright fluorescence; head gland is in greenish grey. **C**, three-dimensional model of acetabular glands; Cy3-azide and Alexa Fluor^® ^488 stained cercaria was employed for the reconstruction; postacetabular glands are in green, circumacetabular glands in red; wide arrow shows acetabulum, thin arrow points to the strangulation of duct bundles entering muscular conus of the head organ (also see Additional File 1). **D**, visualization of cercarial circumacetabular glands by alizarin and fluorescence microscopy; cercaria was anaesthetized by Procain, acetabulum is exserted; marked are the posterior and anterior circumacetabular gland cells on the right side of cercaria. **E**, combination of autofluorescence and staining by FITC-phalloidin of the cercaria in CM; arrow points to the area where gland ducts enter the muscular conus; projection series. **F**, three-dimensional model of cercarial head gland; lobated cercarial head gland (in dark blue) with the three bundles of acetabular gland ducts on each side running through the head gland cell within the head organ (each bundle coloured separately). **HG**, head gland; **dPA**, ducts of postacetabular glands; **CA-p **and **CA-a**, posterior and anterior circumacetabular cells, respectively; **PA **and **CA**, postacetabular and circumacetabular glands, respectively; **MC**, muscle conus; **A**, acetabulum.

A schematic figure shows the distribution of glands within the body of *T. regenti *cercaria (Figure 1A). We found that Alexa Fluor^® ^488 binds to secretory vesicles of postacetabular (PA) glands, enabling to distinguish particular vesicles using confocal microscopy (CM) (Figure 1B). Moreover, Cy3-azide stained the cercarial body red but it did not mark any type of glands included in this investigation. Therefore, circumacetabular (CA) glands and head gland (HG) appeared as greenish grey and dark spaces on red background, respectively.

Combined results obtained from CM and transmission electron microscopy (TEM) of *T. regenti *cercariae showed that the three pairs of PA gland cell bodies are located strictly posteriorly from the ventral sucker and occupy significant part of cercarial hind body (Figure 1C). Particular cells can partially overlap, so that the anterior part of a rear cell covers the posterior part of the fore cell. From the anterodorsal part of each cell a cytoplasmic projection protrudes proximally, forming a gland duct filled with secretory granules proceeding from the cell body. Two bundles, each containing a triplet of PA ducts, form on each side of cercarial body (Figure 1C and Additional File 1).

The posterior pair of CA gland cells is located in part behind the ventral sucker (acetabulum), sometimes being partially covered by the most anterior pair of postacetabular cells (Figure 1C). This pair of CA cells traces the dorsal relief of acetabular musculature and touches the anterior pair of CA cells approximately in the middle of the sucker. The anterior pair traces the opposite side of acetabular musculature. Thus, these cells circumfuse about the base of the ventral sucker and should be termed as circumacetabular rather than preacetabular. This description applies to the situation when the acetabulum is inserted into cercarial body (Figure 1C and Additional File 1). As cercaria exserts the acetabulum, CA cells expand medioventrally within the cercarial body (Figure 1D). However, the shape and actual position of the gland cells is significantly affected by the movements of the sucker as well as the whole well-musculated cercarial body.

**Figure 2:**
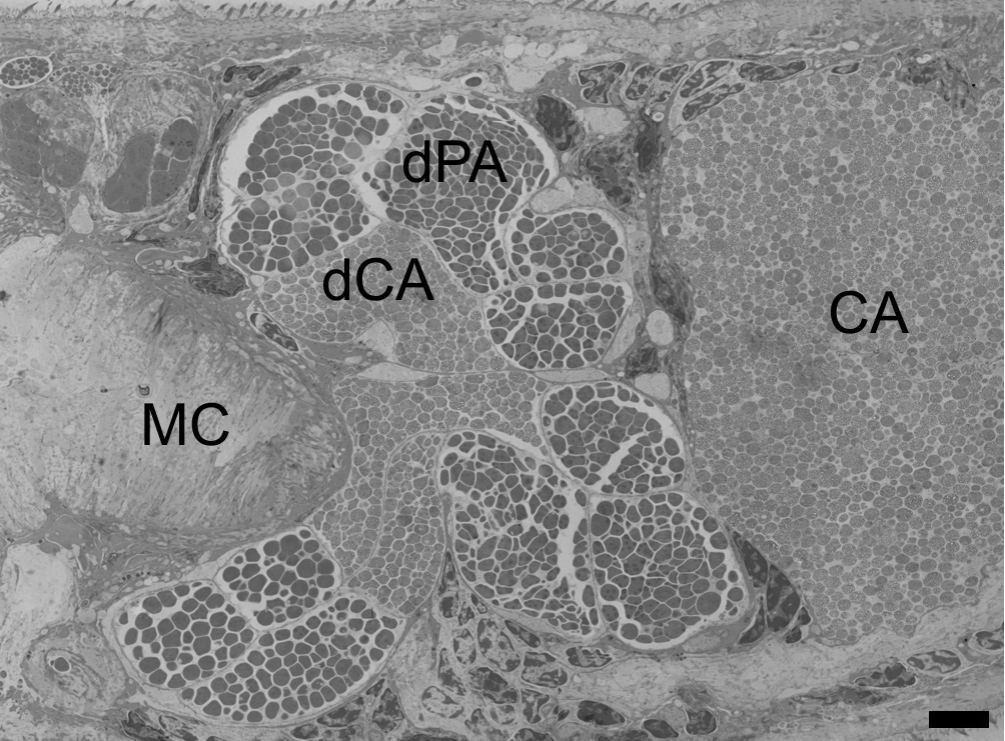
**Formation of duct bundles anteriorly from the acetabulum in *T. regenti *cercaria**. Longitudinal section in TEM; ducts of postacetabular and circumacetabular glands are grouped in two lateral bundles which are in a close contact at this point. **CA**, anterior pair of circumacetabular cells; **dPA**, postacetabular ducts; **dCA**, circumacetabular ducts; **MC**, posterior end of the muscular conus of the head organ; scale bar = 5 μm.

The two protruding triplets of PA gland ducts cover dorsal surface of CA cell funduses. Both the PA ducts and CA cell bodies are in a close contact. On each side of cercaria, a pair of CA ducts joins the triplet of PA ducts in front of the acetabulum; so, two bundles are formed at this level, each containing three PA and two CA gland ducts (Figure 1E and Figure 2). Further on, the two bundles separate from one another and go laterally while turning ventrally, reaching the most ventral position approximately at the level of cercarial eye spots. From this point they turn to a dorsomedian location reaching the surface of the muscular conus of the head organ (Figure 1E). Here the bundles of ducts form a sharp turn in ventral direction, enter the conus lateroventrally and narrow down being strangulated by the musculature (Figures 1C and 1E and Additional File 1). Within the head organ, they form another wave and run through foldings of HG (Figure 1F and Additional File 2).

**Figure 3:**
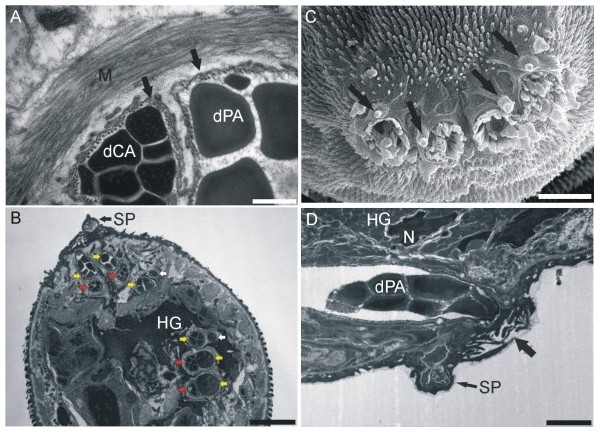
**Electron micrographs of *T. regenti *gland ducts and openings**. **A**, TEM of postacetabular and circumacetabular ducts reinforced by microtubules (arrows) and surrounded by common muscular layer; scale bar = 500 nm. **B**, TEM of the section through the apical part of cercarial head organ; yellow arrows show postacetabular ducts, red arrows show circumacetabular ducts and white arrows point to putative ducts of the head gland; note a sensory papilla adjoining with the gland duct opening covered by tegumental folds; scale bar = 5 μm; (also see Additional File 2). **C**, detail of four gland openings on the head organ of cercaria viewed by SEM; arrows point to sensory papillae in the proximity of the openings; scale bar = 5 μm. **D**, detail of a postacetabular gland duct in TEM leading to surface opening covered by tegumental folds (wide arrow) on the apex of cercaria, sensory papilla in the proximity of gland duct opening is pointed by thin arrow; scale bar = 2 μm. **M**, muscular layer; **dCA **and **dPA**, ducts of circumacetabular and postacetabular glands, respectively; **SP**, sensory papilla; **HG**, head gland; **N**, nucleus of the head gland.

Particular ducts are stiffened by submembrane longitudinal microtubular layers from the site of joining in paired bundles. Each pentad of ducts forming a bundle is also surrounded by circular musculature (Figure 3A). While running through HG, each of the two bundles splits into three - two containing one PA plus one CA duct, and one containing a single PA duct plus a joining duct of HG (Figure 3B). In the most apical part of the head organ, the single PA duct joins one of the bundles composed of 1 PA + 1 CA ducts. The four resulting bundles (two on each side) come to four openings located slightly subterminally on the apex of cercaria (Figure 3C), from which secretory granules are released upon stimulation. The openings are covered by several layers of tegumental folds forming a barrier between the lumen of ducts and outer space (Figures 3B and 3D). Each of the openings is connected with a major sensory papilla; additional papillae also occur around the openings (Figures 3B-3D).

HG of *T. regenti *cercaria is located entirely within the muscular sac of the head organ. Evaluating serial sections, it seems to be composed of one cell only with a nucleus located submembraneously (Figure 3D). This cell has a complicated shape; its fundus projects into various lobes enclosing bundles of penetration gland ducts and muscle cell nuclei within the head organ (Figure 3B). The structure is more lobed in the anterior part from which also main projections proceed to the apex of the head organ (Additional File 2).

### Ultrastructure of glands and secretory vesicles

All three types of gland cells are filled with secretory vesicles, which differ in size, shape and general appearance in TEM. The features of vesicles in particular gland types seemed to depend mainly on the method of fixation used and further sample treatment.

**Figure 4:**
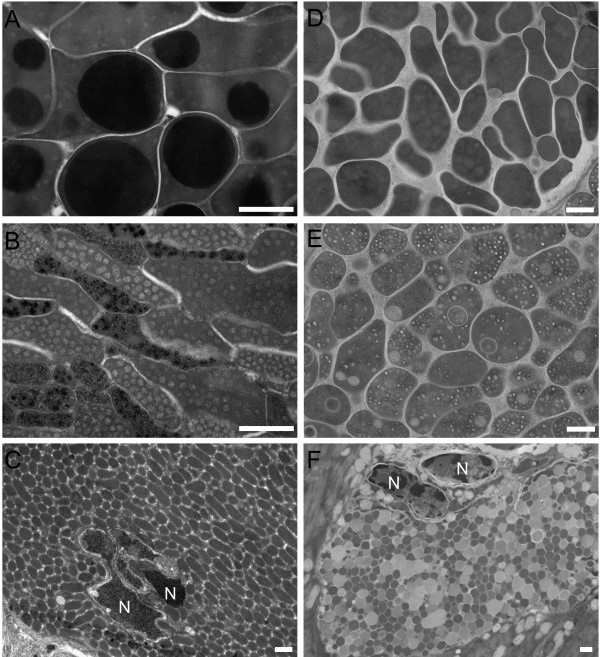
**Details from transmission electron microscopy of secretory vesicles from particular glands of *T. regenti *cercaria**. **A-C**, "classical" method of sample treatment; **D-F**, high-pressure freezing/freeze substitution method. **A+D**, postacetabular vesicles. **B+E**, circumacetabular vesicles. **C+F**, vesicles in the head gland. **N**, nuclei of muscle cells surrounded by the head gland cell. Scale bars = 500 nm.

Using the "classical" method of fixation and embedding into Spurr resin, PA vesicles were mostly irregular in shape, their length varied between ca. 1-2 μm when sectioned in longitudinal median plane. They contained one or few homogenous globular electron-dense cores within a less electron-dense matter, which was slightly granular and usually appeared more electron-dense than that of CA vesicles (Figure 4A), although this feature varied among particular samples. These cores varied in size and filled significant part of the vesicles.

The vesicles in CA gland cells were somewhat smaller than those in PA cells and more elongated in shape. Their content, also slightly granular, was usually more electron-lucent (variations occurred, too). Numerous spherical granules were observed within the vesicle matter. Their size was up to 100 nm. The structure of these granules was not homogenous and their electron density varied among vesicles, but each particular vesicle contained only one type of the granules (Figure 4B). The vesicles within both acetabular gland types (PA and CA) are tightly conglobated, embedded in a thin granular matrix. The size of secretory vesicles increases in the most distal parts of gland ducts (Figure 3D).

The content of HG was composed of more or less uniform oval vesicles ca. 0.4 - 0.6 μm long, filled with homogenous granular content of moderate electron density. The space among vesicles was occupied by thinner granular matrix (Figure 4C).

Alternatively, employing high-pressure freezing and freeze substitution for TEM specimen preparation, vesicles in all three gland types retained more or less globular shape. PA vesicles did not harbour large electron-dense granules observed in case of the "classical" method of fixation and embedding. Instead, they contained delimited spherical granules which were slightly less dense than gland matrix, although a smaller proportion of vesicles possessed even almost electron-lucent granules (Figure 4D).

CA vesicles contained numerous small electron-lucent granules and in many vesicles larger spherical bodies could be observed, containing subtly granular matter (Figure 4E). The vesicles in both acetabular gland types were embedded in fine granular matrix.

The vesicles within HG were heterogeneous in terms of their electron density. At least two distinct types could be observed: 1) larger vesicles possessing moderately electron dense finely granular matter and 2) smaller but more electron-dense finely granular vesicles (Figure 4F).

### Estimations of gland volumes and pH

The volumes of particular gland types (including ducts) relative to the volume of whole cercarial body (excluding tail) were determined by means of stereological method on sections of cercariae in TEM. PA glands occupied 14.4%, CA glands 12% and HG 6% of the body volume (Table 1). Mean total volume of cercarial body obtained from optical sections of 20 lectin-labelled cercariae was 487415 μm^3 ^± 21.3%.

**Table 1:** Results of the estimations of relative and absolute volumes of cercarial body and glands

	TEM + CM stereology	CM stereology	CM voxel counting
**Postacetabular glands**	14.4%	19.5% ± 3.3%	18.7% ± 5.4%
	70065 ± 4491	94114 ± 3106 (fixed)	107372 ± 5798
		136570 ± 4507 (live)	

**Circumacetabular glands**	12%	12.5% ± 3.5%	15.2% ± 5%
	58255 ± 3734	60330 ± 2112 (fixed)	87275 ± 4364
		87545 ± 3064 (live)	

**Head gland**	6%	ND	ND
	29258 ± 1875		

**100% body (absolute volume)**	487415 ± 31240 (fixed)	482637 ± 159753 (fixed)	574180 ± 197518 (fixed)
		700360 ± 158982 (live)	

Columns represent particular methods used for estimations. Relative volumes are indicated as percent of total volume of cercarial body (excluding tail). Absolute volumes are expressed in μm^3 ^± standard deviation. TEM, sections under transmission electron microscope; CM, optical sections under confocal microscope; ND, not determined.

Confocal microscopy of fixed or anaesthetized labelled cercariae and their acetabular glands was used to confirm correctness of volume estimations by the previous method. HG was not included in these experiments. The average volume of the whole cercarial body seemed to be significantly affected by fixation process. The value for live anaesthetized cercariae was 700360 μm^3 ^± 22.7% comparing to 482637 μm^3 ^± 33.1% of fixed cercariae (Table 1). Nevertheless, relative volumes of glands (expressed as percentage of total body volume) were strikingly in accord when comparing both types of material. Using a voxel counting method, the relative volume of PA glands was 18.7% ± 5.4% (n = 9) and of CA glands 15,2% ± 5.0% (n = 3). Stereological method showed relative volumes 19.5% ± 3.3% (n = 9) for PA glands and 12.5% ± 3.5% (n = 9) for CA glands (Table. 1).

The estimations of pH in acetabular glands using SNARF-1 pH-dependent fluorescent probe showed that the value in CA glands was higher (7.44 ± 1.06) than in PA glands (7.08 ± 0.86).

## Discussion

The number of penetration glands and their ability to be stained by various dyes have already been shown previously for *Trichobilharzia regenti *cercariae [5,11]. In this study, we used some other techniques for fluorescent visualization of acetabular glands which are applicable in confocal microscopy, namely labelling by alizarin and Alexa Fluor^® ^488.

The ability of alizarin to stain CA gland content in schistosome cercariae has been previously exploited in light microscopy - e.g. [1]. It is capable of marking structures containing calcium; this element has been shown to occur in CA glands of *Schistosoma mansoni *in a high concentration [14], most likely in an ionic form [13]. However, it is known that while being a weak complex-forming agent with respect to the free Ca^2+ ^ions, alizarin exhibits affinity to calcium in biological structures, forming heteroligand complexes with biological objects [32]. Similarly to *S. mansoni*, the presence of a significant amount of calcium in CA glands of *T. regenti *was proven previously [11]. Thus, we employed alizarin for visualization of CA glands in confocal microscopy as it produces fluorescence upon excitation at 488 nm (see Figure 1D). Cercariae had to be alive because alizarin stains the glands intravitally and starts to leak out of fixed specimen. For this reason we immobilized cercariae by Procain anaesthesia. This was previously used for the purpose of photography of live cercariae in fluorescence microscopy [33]. However, immobilization is neither permanent nor total, which is problematic for z-scanning using a confocal microscope. Therefore, the number of completely scanned cercariae was not high. Nevertheless, we confirmed that the four gland cells described originally as preacetabular occupy in fact the space around the acetabulum; therefore, they should be called rather circumacetabular glands.

During investigations of cercarial musculature [34] by labelling with FITC-phalloidin for actin filaments we found that acetabular gland cells express autofluorescence. Besides this, various fluorescent probes have been tested to visualize the glands and secretory vesicles - Alexa Fluor^® ^488 was the most useful one. All the approaches mentioned above were successfully used for 3-D reconstruction of gland shapes or for estimation of gland/cercarial body volumes.

Recently, Collins et al. [35] published their work on labelling of *S. mansoni *cercarial glands using fluorescent lectins [35]. Comparing our three-dimensional model of *T. regenti *cercarial glands with *S. mansoni *[7,8,35] showed that not only the number and types of glands are common features of both schistosomes, but also the organization, shape and gland openings look very similar. The authors [35] mentioned the previous work of Dorsey and Stirewalt and Dorsey et al. [7,10] who estimated that *S. mansoni *glands occupy nearly two thirds of cercarial body volume; unfortunately, none of these three papers brought exact data based on measurements. We believe that their estimation based just on visual observation was very inaccurate as our initial supposition for *T. regenti *relative gland volumes was also higher than the results of stereometry (one third of the body volume).

We confirmed that there are four visible openings on the apical surface of cercaria from which the contents of acetabular glands are released. Our data enable us to suppose that it is highly unlikely that the number of the openings may vary depending on branching of gland ducts outside bundles, as mentioned by these authors for *S. mansoni*. Each bundle containing both PA and CA ducts is enveloped by a common muscle layer which supposedly controls release of secretory vesicles. Therefore it is probable that both gland types are released together during penetration into host skin. This is in accord with the hypothesis that the two separated compartments (PA and CA secretory cells) contain substances which may be activated after mixing of their contents outside cercarial body [11]. However, we are aware of *in vitro *experiments in which sequential emptying of particular penetration gland types was observed [36]. This process certainly deserves further research.

Some doubts have existed about the architecture of the head gland in *S. mansoni *cercaria. It was pointed out that it might be composed of two cells or at least contain two nuclei [10], although this statement was rather speculative. During our experiments we have not found any evidence that there could be two cells composing the head gland in *T. regenti*.

The features of secretory vesicles produced by particular gland types are markedly affected by the mode of material treatment for TEM. From this point of view, high-pressure freezing and freeze substitution method gave the best results and more structural details could be evaluated. In general, it can be stated, that the vesicles in *T. regenti *glands resemble those in corresponding gland types of *S. mansoni *(reviewed by Dorsey et al. [10]). A diference was seen in size of the vesicles which were 2-3 times larger in case of *T. regenti*. This is probably related to the body size differences between *T. regenti *(total length ca. 760 μm [5]) and *S. mansoni *cercaria, which is smaller (ca. 500 μm; [10]). Although the composition of acetabular glands of *T. regenti *seems to be different to some extent from that of *S. mansoni *(e.g. in terms of types of proteolytic enzymes, presence of mucous glycosubstances [11,22,28,30]), this obviously has a little effect on the appearance of secretory vesicles in TEM. In CA vesicles of *T. regenti*, similar electron-lucent granules were observed as in *S. mansoni*, to which calcium ions were localized [13]. This is another (indirect) proof of the presence of calcium in CA glands of *T. regenti *which may have consequences in interpretation of calcium function in these gland cells. It was confirmed, that the role of Ca^2+ ^could be in regulation of proteolytic (serine peptidase - cercarial elastase) activity within (or outside) CA glands [37,38]. However, it has been proven by various methods that *T. regenti *does not possess this enzyme and instead it most likely uses cysteine peptidases (e.g. cathepsin B2) localized in PA glands [11,28,30,39].

The three approaches to estimate volumes of cercarial glands gave similar results. Both fixed and live cercariae were used in these experiments to compare the effect of fixation on the shrinkage of cercarial bodies and of gland cells. The total volumes of fixed cercarial bodies decreased to ca. 69% of that of live anaesthetized cercariae, but the relative gland volumes remained similar in both cases, ie. between 14.4% - 19.5% for PA glands and 12% - 15,2% for CA glands, with low variations depending on the method used. Relatively high standard deviations were caused by natural variability among individual cercariae and probably also by their eminent contractility. In the case of voxel counting method for evaluation of optical sections obtained by confocal microscopy, there may be a little over- estimation due to inappropriate image enhancement processing antecedent to volume estimation. The estimation of head gland relative volume was 6% using TEM and stereology; we did not evaluate its volume by confocal microscopy as we have not succeeded in finding a specific fluorescent marker for this gland. All three gland types together fill around 1/3 of cercarial body which stresses the importance of these secretory cells for the cercarial stage of schistosomes employing their secretions during invasion of vertebrate hosts. These data will help in the future to estimate quantities of bioactive molecules found within the glands of *T. regenti*. Unfortunately, no volumetric data exist from *S. mansoni *model, making exact comparisons impossible. The volumes could be just adequate to the size of cercarial body, which is about 1.5 × longer than that of *S. mansoni *cercaria [5,10]. But, supposedly, the relatively high volume of gland contents enables the cercariae of bird schistosomes to penetrate through the tough skin on exposed duck legs. Also, it may be the cause of higher invasion efficiency by *Trichobilharzia *compared to *Schistosoma mansoni *in the case of human skin [40].

The estimation of pH inside acetabular glands of *T. regenti *was quite a difficult task. Although fluorescent probes like SNARF-1 expressing shift in the wavelength of emitted light depending on pH are commonly used for pH estimations in cells or their compartments (e.g. [41]), in our experiments it was necessary for this compound to cross the cercarial glycocalyx, syncytial neodermis including its basal lamina and cell membranes of gland cells. For the purpose of calibration of the system by a set of buffers of known pH, nigericin was used as H^+ ^ionophore to disrupt pH gradients. This might to some extent influence the real picture about the actual pH within the glands. However, at least a tendency could be seen of higher pH in CA comparing to PA glands. This result was anticipated as CA cells contain a relatively high amount of Ca^2+ ^cations - their anionic counterpart is not exactly known but it could be hydrogen carbonate and carbonate in *S. mansoni *according to [42]. Surprisingly, we recorded higher pH in PA cells than expected. It was shown that PA glands produce a cysteine peptidase (cathepsin B2) which has an optimum of activity at pH 6.0 [30]; its activity drops dramatically at pH above 6.5. Therefore it can be hypothesized, that the higher value of pH in PA cells (around neutral) could restrain the hydrolytic activity of cathepsin B2 in order to prevent autolysis of cercaria. The activity of this acid-active peptidase should be restored upon the contact with the stratum corneum of the skin, which has pH usually between 4 - 6.5 in humans [43] and 5.4 - 6 in domestic ducks [44] due to the presence of organic acids forming an antimicrobial protective barrier ("acid mantle") in the upper layer of vertebrate skin.

## Conclusions

The ultrastructure and architecture of cercarial gland apparatus of *Trichobilharzia regenti *resemble that of the human parasite *Schistosoma mansoni*, although minor differences could be found in size and appearance of secretory vesicles. Different chemical composition of *T. regenti *glands does not seem to affect ultrastructural features of the vesicles. The new protocols for gland and vesicle visualization introduced in this study which will be useful for further research of host invasion by schistosome cercariae.

The measurements of volumes of particular gland assemblages of *T. regenti *and 3-D modelling of the glands performed for the first time in a cercaria will help in quantification of known enzymatic activities within the glands in order to better understand the ability of this neuropathogenic parasite to invade non-specific mammalian hosts.

The first estimations of pH values in acetabular glands of *T. regenti *cercariae can help our understanding of the physico-chemical environment, which most likely affects enzymatic activities within the gland cells of schistosomes and other features of gland secretions.

## Methods

### Parasite

The life cycle of *Trichobilharzia regenti *Horák, Kolářová et Dvořák, 1998 has been maintained in the Department of Parasitology, Faculty of Science, Charles University in Prague using domestic ducks (*Anas platyrhynchos *f. dom.) and *Radix lagotis *snails as definitive and intermediate hosts, respectively. Cercariae were shed from experimentally infected snails exposed to light in small beakers and collected from the water surface.

### Gland architecture

Three-dimensional models and reconstructions of cercarial glands were based on CM. Cercariae were fixed with 2% formaldehyde for 10 min, rinsed in water, incubated in 0.2% Triton-X100 for 5 min, rinsed in water, incubated in the solution of 20 μM Cy3-azide and 20 μM Alexa Fluor^® ^488 (Invitrogen) in PBS for 30 min, washed in PBS and images of optical sections were acquired by confocal microscope Zeiss 5 DUO (Carl Zeiss Inc.) with following settings: excitation - 488 nm and 560 nm, emission - 505-550 nm and 575 nm for Alexa Fluor^® ^488 and Cy3 respectively. The compartments of interest were reconstructed using Amira software.

### Ultrastructural studies

The ultrastructure of cercarial glands was studied by means of TEM. The "classical" method was performed as follows: collected cercariae were fixed with 2.5% glutaraldehyde in 400 mM Hepes buffer supplemented by 150 mM NaCl overnight at 4°C, posfixed in 1% OsO4 and dehydrated in a graded ethanol series terminated with 3 washes in 100% acetone. Then, cercariae were embedded in Spurr resin (Polysciences Inc.). Blocks were sectioned to 60-70 nm. The sections were conventionally contrasted with uranyl acetate (3%) and lead citrate (0.1%) and examined under JEOL 1011 microscope.

For high-pressure freezing and freeze substitution, cercariae were dropped into to 20% bovine serum albumin (BSA) and frozen in the Leica EM PACT high-pressure freezer [45]. Frozen samples were transferred under liquid nitrogen to the Leica AFS machine and placed in the substitution solution pre-cooled to -90°C. The freeze substitution was performed in acetone containing 2% OsO_4_. The samples were embedded in Epon resin (Spi-Chem).

For scanning electron microscopy, suspension of cercariae was dropped into hot buffered 4% formaldehyde and left for 2 hours at room temperature, dehydrated in a graded ethanol series terminated with 3 washes of 100% acetone. Dehydratation was completed in the Critical Point Dryer (CPD 030, BAL-TEC). Finally, cercariae were arranged on a metal target with carbon tape, gold-coated in Sputter coater (SCD 030) and examinated under JEOL 6380LV microscope.

### Estimation of gland volumes

The estimations of relative volumes of postacetabular, circumacetabular and head glands (related to cercarial body volume) were performed independently employing two approaches - TEM and CM, both supported by stereological methods. Results were compared.

For TEM and stereology, cercariae were fixed in a mixture of 2% glutaraldehyde and 0.05M CaCl_2 _in 0.1 M cacodylate buffer and postfixed in 1% OsO4 in 0.1 M cacodylate buffer, pH 7.2. Dehydration was performed in a graded ethanol series and propylenoxide, embedding in Epon resin. Sections 70 nm thin were stained with 3% uranyl acetate and viewed with electron transmission microscope Morgagni 268 (FEI Company) equipped with Megaview II camera. Relative volumes of particular glands were deduced from the electron microscopy images. The volume was calculated from the ratio of relative areas occupied by individual glands and the total area of cercarial body on TEM sections. Two hundred sections from five Epon blocks each containing around 20 cercariae were evaluated during this analysis. The square lattice of regularly spaced points was used for the area estimation (see e.g. [46]). The total volume of *T. regenti *cercaria was estimated by means of Cavalieri's method [46] from optical sections of twenty cercarial bodies fixed in 2% formaldehyde (excluding tails) and labelled with a fluorescein-conjugated fucose-specific lectin (*Ulex europaeus*-I, Vector laboratories) [33]. The FITC/lectin conjugate stained exclusively surfaces of cercariae. Absolute volumes of particular glands were estimated using the values of relative gland volumes and mean total body volume.

For the estimation of gland volumes based on CM, various staining procedures were used. PA glands expressed significant autofluorescence during labelling of cercarial actin filaments by FITC-coupled phalloidin [34]. CA glands were visualized by staining living cercariae in 0.5% aqueous solution of alizarine (Sigma) for 2 min. Parasites were immobilized in 0.5% aqueous solution of procainum hydrochloricum anaesthetics (Procain, Léčiva Praha) and mounted in Vectashield medium. The samples with fixed or immobilized stained cercariae were observed using confocal microscope Leica TCS SP2 (Leica Microsystems, Germany). Due to remarkable autofluorescence of PA glands, autofluorescence multispectral imaging into several channels was performed. For each object, several optical sections have been obtained to cover the whole object volume. In the case of living immobilized cercariae stained with alizarine, three different channels were captured. These were: transmission channel with differential contrast, reflection channel to obtain better information about the whole area of cercarial section in focal plane and the channel with alizarine excited with 488 nm laser and emmision spectra collected in a range between 498 and 800 nm.

Two methods have been performed for the volume calculation of both PA and CA glands in CM. The voxel counting method was performed on binary images, where the whole glands were segmented using several image enhancing methods (like mean, median, Gaussian etc. filtering and their combinations) until the threshold could be set up with sufficiency according to visual accuracy. The areas in each optical section were measured and multiplied by respective voxel height and summed together, including all sections. The obtained values represented the estimated absolute volumes of cercarial bodies or glands. Finally, the percentage of the gland volume was evaluated as the ratio of respective absolute volumes. For more precise volume estimation we used a stereological method [47,48] implemented into the ImageJ software plug-in called VolumEst [49]. Here we used the "object type" parameter "irregular" and a square grid width 25 μm point distance.

### Estimations of pH within glands

The estimations of pH were performed for both postacetabular and circumacetabular glands. Three independent experiments were performed, in each 20 cercariae were analyzed. Cercariae were loaded with 10 μM SNARF-1 acetoxymethyl ester (Invitrogen) for 1 h at RT, rinsed with tap water and incubated in the tap water for additional 30 min at room temperature to allow complete hydrolysis of ester bonds. The objects were embedded into 2% low melting agarose type IX (Sigma, gelling at 8-17°C), 50 μl of the agarose mixture with cercariae was transferred on coverglass in 35 mm Glass Bottom Microwell Dishes (MatTek Corporation) and covered with coverslip; dishes were incubated on ice for 5 minutes. The couples of fluorescence images were acquired with confocal microscope (Zeiss 5 DUO, Carl Zeiss, Inc.) with following settings: excitation - 514 nm, emission - 560-600 nm (band F1) and 620-660 nm (band F2), pinhole - 896 μm. The ratios of images (F2/F1) were produced by the division of corresponding pixels in the image pair using the ImageJ software. The ratio values were calculated as the average pixel intensity of values extracted from areas corresponding to PA or CA glands. *In situ *calibration was performed using the set of buffers containing 120 mM KCl, 30 mM NaCl, 0,5 mM MgSO4, 1 mM CaCl_2_, 5 mM glucose, 10 mM Hepes and 50 μM nigericin - pH of particular buffers was 6.008, 6.571, 7,006, 7.540 and 8.010. The 3 h incubation in nigericin buffer was necessary for the total collapse of pH gradient. The ratio of intensities was fitted to the exponential growth regression described by the equation: *y *= *a *× *e*^[bx]^, where *y *is equal to the ratio of intensities, *x *to the value of pH. Constants *a *and *b *were calculated using SigmaPlot software.

## Ethical approval details for use of experimental animals

All experiments and the maintenance of experimental animals were consistent with current animal welfare laws of the Czech Republic and were approved by the Animal Welfare Committee of the Charles University in Prague.

## Competing interests

The authors declare that they have no competing interests.

## Authors' contributions

AL prepared freeze-substitued cercariae, performed studies on the ultrastructure of freeze substituted glands, prepared material for confocal microscopy analysis of stained cercariae, prepared 3-D models including videos and wrote a part of the Methods section. JB performed TEM studies on the ultrastructure of glands, prepared material for confocal microscopy, contributed to conception, participated in interpretation of data and wrote a part of the Methods section. OS performed confocal microscopy for estimations of gland volumes, analyzed data, made calculations and wrote a part of the Methods section. MK participated in interpretation of data and revised the manuscript critically for important intellectual content. KK performed stereological analysis using optical and epon sections, performed experiments focused on the estimation of pH of secretory glands including the analysis of the received data and wrote a part of the Methods section. LM prepared conception, coordinated experiments, participated in interpretation of data, prepared material for confocal microscopy of glands and wrote the manuscript. All authors read and approved the final manuscript.

## Supplementary Material

Additional file 1 **Three-dimensional model of acetabular penetration glands of the cercaria of *Trichobilharzia regenti***. Postacetabular glands are in green, circumacetabular in red. Bundles of all ducts are in green for simplification (also see Figure 1C). Click here for file

Additional file 2 **Three-dimensional model of the head gland of *Trichobilharzia regenti *cercaria with the three bundles of acetabular gland ducts penetrating on each side**. Lobated cercarial head gland is in dark blue. Acetabular duct bundles are coloured separately (also see Figure 1F). Click here for file
